# Poor long‐term cardiovascular risk factor management after acute coronary syndrome: An observational cohort study

**DOI:** 10.1111/joim.70086

**Published:** 2026-03-20

**Authors:** Jessica Schubert, Maria K. Svensson, Margret Leosdottir, Bertil Lindahl, Håkan Melhus, Elin Täufer Cederlöf, Nina Johnston, Thomas Cars, Emil Hagström

**Affiliations:** ^1^ Department of Medical Sciences Uppsala University Uppsala Sweden; ^2^ Uppsala Clinical Research Center Uppsala Sweden; ^3^ Department of Clinical Sciences Malmö Lund University Malmö Sweden; ^4^ Department of Cardiology Skane University Hospital Malmö Sweden; ^5^ Sence Research AB Uppsala Sweden

**Keywords:** acute coronary syndrome, secondary prevention, target assessment

## Abstract

**Background:**

Cardiovascular risk factor control reduces the risk of recurrent events after acute coronary syndrome (ACS). Long‐term post‐ACS management is poorly studied.

**Objectives:**

To assess frequency of low‐density lipoprotein cholesterol (LDL‐C) and systolic blood pressure (SBP) monitoring and target achievement during 3 years post‐ACS.

**Methods:**

Data from all patients hospitalized for ACS in Uppsala between 2012 and 2020 were obtained from electronic health records (EHRs). The probability of measurement and target achievement was estimated with adjusted regression models. Associations were assessed for sex, participation in cardiac rehabilitation (CR), presence of diabetes with nurse‐led follow‐up and documentation of an ICD‐10 code for chronic ischaemic heart disease in the EHR.

**Results:**

Among 6083 patients (median age 73 years, 34% female), follow‐up data were available for 72% in year 1, 56% in year 2 and 44% in year 3. Year 3 LDL‐C was not measured in 48% of patients, and 19% were at target (<1.8 mmol/L). For SBP, 16% lacked a measurement, and 50% were at target (<140 mmHg). Female sex was associated with lower probability of having LDL‐C measured or having LDL‐C or SBP at target. CR participation, structured diabetes follow‐up or having a chronic ischaemic heart disease code in the EHR was associated with a higher probability of LDL‐C and SBP measurements at 2 and 3 years.

**Conclusions:**

Lack of measurement and low risk factor achievement highlight missed opportunities in secondary prevention. Female sex, lack of structured follow‐up and the absence of diagnostic labelling were associated with less frequent monitoring and poorer risk factor control.

## Graphical abstract

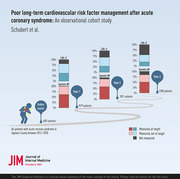

## Introduction

Optimal secondary prevention, including lifestyle and pharmacological interventions, reduces the risk of future atherosclerotic cardiovascular events and death for patients after acute coronary syndromes (ACSs) [1]. Secondary prevention is most effectively provided through comprehensive, structured cardiac rehabilitation (CR) programmes [2]. Still, many post‐ACS patients remain undertreated, leaving them at increased risk of cardiovascular events and death.

Despite proven benefits, most post‐ACS patients do not achieve guideline‐directed standards for secondary prevention [3, 4, 5]. In Sweden, the majority of patients with myocardial infarction (MI) are offered participation in a structured, comprehensive and supervised CR programme during the first year post‐MI [6]. The programme generally consists of several follow‐up visits to a nurse, physician and physiotherapist and offers supervised centre‐based exercise training and group‐based patient education [7]. The majority of patients attending CR in Sweden reach cholesterol and blood pressure targets during the first year post‐MI, the proportion increasing steadily during the last two decades [8] and comparing favourably to international results. However, there is still room for improvement, and a large proportion of these patients have high residual risk of recurrent atherosclerotic cardiovascular events and death [9].

After the first year with centre‐based CR, patients are referred to primary care for continued secondary prevention. Further, due to lack of centre‐based CR resources in Sweden, many patients above 80 years of age and those with severe comorbidities are referred to primary care directly after the ACS hospitalization. Although centre‐based CR interventions and results are closely monitored through the nationwide SWEDEHEART registry, the same does not hold true for follow‐up in primary care. Consequently, evidence on measurement frequency and target achievement after the first year post‐ACS remains limited.

The aim of this study was to evaluate the monitoring frequency and control of low‐density lipoprotein cholesterol (LDL‐C) and systolic blood pressure during the first 3 years after an ACS, patterns of lipid‐lowering medication prescriptions, and to assess whether outcomes differed according to sex, participation in centre‐based CR during the first year post‐ACS, presence of diabetes, which in Sweden is systematically followed through structured nurse‐led care [10, 11], or documentation of chronic coronary syndrome (I25) in the electronic health record (EHR), based on the hypothesis that this diagnosis signals increased risk to the treating physician.

## Methods

### Study design and setting

This was a retrospective observational cohort study of all patients hospitalized for ACS in Uppsala County, Sweden between January 2012 and December 2020. Patients were followed for up to 3 years or until death, recurrent ACS or loss to follow‐up. Uppsala county has approximately 350,000 inhabitants. Since 2005, a unified EHR has been implemented across all private and public primary care centres and hospitals, enabling complete longitudinal capture of healthcare conducted within the region.

### Data sources

Longitudinal data on LDL‐C and systolic blood pressure measurements, target achievement and lipid‐lowering medication prescriptions were automatically extracted from the EHR covering all healthcare within the county. Further, diagnosis codes (ICD‐10), laboratory measurements, clinical measurements, other relevant prescriptions and healthcare visits were extracted (Table S1).

Tobacco use codes (Z72.0, F17.2) were available; however, smoking status was not entered in a structured fashion and was therefore excluded from analyses.

### Study population and follow‐up

All patients ≥18 years of age with a primary discharge diagnosis of unstable angina or MI (ICD10 codes I20.0 or I21–I24) admitted at either of the two acute cardiology care units in Uppsala County were eligible. The index event was defined as the first recorded ACS hospitalization during the study period. Follow‐up continued until a new ACS, death, relocation from the county or study end (31 December 2020), at which point a new observation period began. There were no exclusion criteria. All patients were captured in the joint EHR system.

### Cardiac rehabilitation exposure

Patients were classified as participating in centre‐based CR if they had

1. A CR centre visit to a physician 6–10 weeks after discharge, and
2. A CR centre visit to a physician or a nurse 11–14 months after discharge [6, 7].

Centre‐based CR programmes in Sweden entail routine LDL‐C and systolic blood pressure measurements at both time points. This stratum was compared to patients with at least 14 months available follow‐up not attending centre‐based CR. Patients with only one CR visit were classified as non‐participants, as programme completion requires both early and late follow‐up.

### Clinical definitions and covariates

Comorbidities were identified using ICD‐10 codes and prior medications recorded in the EHR from 2005 and onwards. Definitions included the following:

- Diabetes mellitus Type 1 or 2: at least one of ICD‐10 E10–E14, HbA1c >48 mmol/mol or prescription of glucose‐lowering therapy (ATC A10) prior to index ACS.
- Hypertension: at least one of ICD‐10 I10.9, systolic blood pressure >140 mmHg or diastolic blood pressure >90 mmHg within 3 years before index ACS.
- Congestive heart failure: ICD‐10 I50.
- Chronic obstructive pulmonary disease: ICD‐10 J44.
- Obesity: BMI >30 kg/m^2^ or ICD‐10 E66 within 3 years.
- Revascularization: procedural codes FNA‐H, FNJ‐K or FNW during index hospitalization.

Estimated glomerular filtration rate was calculated using the revised Lund–Malmö equation from plasma creatinine [12]. LDL‐C was directly measured in accredited laboratories subject to regular external quality audits. Systolic blood pressure was recorded in routine clinical practice, with recommendations specifying measurement in a seated position after 5 min of rest using validated manual devices, with repeated measurements if elevated.

During the study period, multiple European guidelines were used with different prevention targets for LDL‐C [13, 14, 15] and systolic blood pressure [16, 17]. For consistency, targets were defined according to the European Society of Cardiology recommendations prevailing for most of the study period as follows:

- LDL‐C <1.8 mmol/L
- Systolic blood pressure <140 mmHg

Chronic ischaemic heart disease was defined as the presence of ICD‐10 code I25 recorded during any healthcare visit within the current follow‐up year.

Prescription of lipid‐lowering therapy was defined as at least one filled prescription per calendar year from ATC classes C10AA or C10AX09, based on the nationwide dispensation database integrated into the EHR. This metric reflects medication access and continued treatment indication but not adherence or treatment intensity.

### Outcomes

Primary outcomes were as follows:

1. the proportions of patients with recorded follow‐up measurements of LDL‐C and systolic blood pressure, and
2. the proportions of patients achieving guideline‐recommended prevention targets for LDL‐C and systolic blood pressure.

Secondary outcomes included median achieved LDL‐C and systolic blood pressure levels.

Outcomes were assessed at three time points as follows:

- year 1: ≤12 months post‐ACS
- year 2: >12 to ≤24 months post‐ACS
- year 3: >24 to ≤36 months post‐ACS

For each year, the last available measurement was used.

### Statistical analyses

Descriptive statistics were reported as counts and proportions for categorical variables and medians with interquartile ranges for continuous variables.

Adjusted probabilities of risk factor measurement and target achievement were estimated, using average marginal effects from logistic regression models [18, 19]. These represent the covariate‐adjusted marginal probability of each outcome, averaged across the observed distribution of covariates.

Adjusted mean LDL‐C and systolic blood pressure levels were estimated using linear regression models, using the covariate set from the logistic regression models. These marginal means represent expected group‐level values adjusted for baseline differences.

The analyses were descriptive in nature and not intended for prediction modelling or risk stratification. Rather, the objective was to characterize associations between clinical exposures and risk factor management in routine care.

Covariates were selected using directed acyclic graphs (DAGs), with one DAG constructed per exposure (Table S2, Fig. S1). Missing data were handled using complete case analysis; no imputation was performed.

For binary outcomes, average marginal probabilities from logistic regression are reported with 95% confidence intervals, and the *y*‐axis in graphical displays represents the probability of the outcome (e.g., measurement performed or target achieved). For continuous outcomes, average marginal means from linear regression are shown with corresponding 95% confidence intervals, indicating the expected average LDL‐C or systolic blood pressure level in each group after adjustment. Outcomes were summarized by year after the index event. Results are presented, using the marginaleffects package in R [20].

Monitoring frequency and risk factor control were assessed across sex, CR participation, diabetes status and an ICD‐10 code for chronic ischaemic heart disease.

The proportion of patients with at least one filled prescription for lipid‐lowering therapy each year was assessed. Patients were stratified by sex and age at diagnosis: <60, 60 to <70, 70 to <80 and ≥80 years.

Analyses were performed at Sence Research AB, Uppsala, Sweden, Uppsala University, and at the Swedish National Infrastructure for Computing at UPPMAX, using R Core Team R Foundation for Statistical Computing, Vienna, Austria, Version 2024.12.1.

The Regional Ethics Committee in Stockholm approved the study in accordance with the Helsinki Declaration (approval numbers Dnr 2019‐06295 and Dnr 2020‐03231).

Language editing was assisted by artificial intelligence tools (Claude, Anthropic), but these were not used for analysis or interpretation of data. All scientific content, analyses and interpretations were independently performed and verified by the authors.

## Results

### Patient characteristics

A total of 6083 patients with ACS were included (Table 1). Median age was 73 years (inter quartile range, IQR 64–81), and 34% were female. At the index ACS hospitalization, LDL‐C was available for 3871 (64%) of the patients with a median LDL‐C of 3.0 mmol/L. When expanding the measurement window to within 1 week of the index diagnosis, 4266 (70%) patients had LDL‐C measurements with a median LDL‐C of 2.9 mmol/L. Systolic blood pressure was available for 6033 (99%) with a median of 140 mmHg. Of all included patients, 31% had diabetes at the time of index ACS, 87% had hypertension and 24% were obese (Table 1).

**Table 1:** Baseline characteristics of included patients.

Variable	All patients	Patients participating in CR	Patients not participating in CR
Number of patients	6083	2214	3869
Age (years)	73 (64–81)	67 (60–73)	77 (68–84)
Female	2082 (34%)	610 (28%)	1472 (38%)
BMI (kg/m^2^)	26.6 (24.0–29.7)	27.5 (25.0–30.8)	26.6 (23.7–29.8)
Systolic blood pressure (mmHg)	140 (125–160)	140 (126–160)	140 (125–160)
Diastolic blood pressure (mmHg)	80 (70–90)	80 (70–90)	80 (70–90)
LDL‐C (mmol/L)	3.0 (2.2–3.8)	3.1 (2.4–3.9)	2.9 (2.1–3.7)
Hypertension	4948 (81%)	1664 (75%)	3284 (85%)
Obesity	1455 (24%)	584 (26%)	871 (23%)
Diabetes	1899 (31%)	622 (28%)	1277 (33%)
Congestive heart failure	1269 (21%)	286 (13%)	983 (25%)
Estimated glomerular filtration rate <60 mL/min/1.73 m^2^	2603 (43%)	570 (26%)	2033 (53%)
Chronic obstructive lung disease	506 (8%)	145 (7%)	361 (9%)
Revascularization during index ACS hospitalization	4067 (67%)	1741 (79%)	2326 (60%)

*Note*: Data are median (interquartile range) and number (%). CR: centre‐based cardiac rehabilitation during the first year after ACS.

Abbreviation: ACS, acute coronary syndrome; LDL‐C, low‐density lipoprotein cholesterol.

The inclusion rate was balanced through the years, around 11% each year and the median follow‐up time was 2.5 (IQR 0.8–5.1) years. Of the included patients, 3642 (60%) were followed until end of study, 1390 (30%) died, 933 (15%) had a new ACS during the study period and 118 (2%) moved out of the county (Fig. S2).

A prior ACS was recorded in the EHR for 1448 (24%) of the patients before study start in 2012 (prior ACS events occurring in other counties would not be captured in these regional EHRs). After the index event, follow‐up data were available for at least 1 year for 4360 (72%) of the patients, for 2 years for 3418 (56%) and for 3 years for 2665 (44%) of the patients. These were patients who survived, remained living in the same county and did not experience another ACS during the period.

### Risk factor assessment and target achievement

**Fig. 1:**
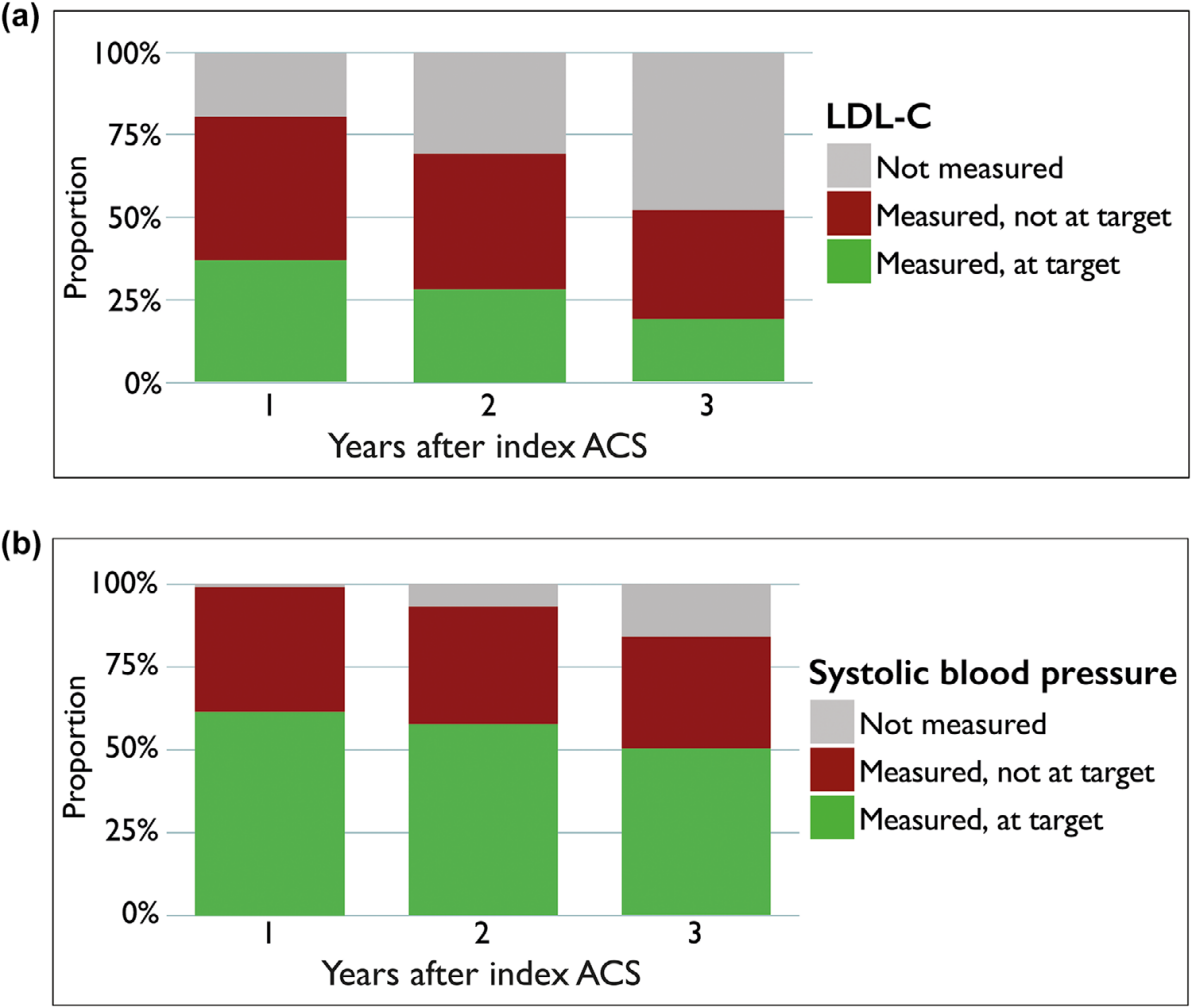
Proportion of patients who had low‐density lipoprotein cholesterol (LDL‐C) (a) and systolic blood pressure (b) measured during the first 3 years after index acute coronary syndrome (ACS) event, and the proportion achieving guideline‐directed target.

During the first year after index ACS, 844 (19%) patients did not have a measurement of LDL‐C. The corresponding numbers were 1044 (31%) and 1270 (48%) for the second and third years after index ACS (Fig. 1a). Of the patients who had an LDL‐C measurement, 1899 (54%) patients did not achieve LDL‐C target during the first year. The corresponding numbers were 1401 (59%) for the second year, and 878 (63%) for the third year after the index ACS (Fig. 1a). Median age and sex distribution are shown in Table S3.

During the first year after the index ACS, 48 (1%) patients did not have a registered measurement of systolic blood pressure. The corresponding numbers were 234 (7%) and 427 (16%) for the second and third years after the index ACS (Fig. 1b). Of the patients who had a measurement, 1631 (38%) patients were not at target systolic blood pressure. The corresponding numbers were 1212 (38%) for the second year, and 900 (40%) for the third year after the index ACS (Fig. 1b).

During the first year after the index ACS, 3798 patients (87%) filled at least one statin prescription, second year 2833 (83%) and third year 2147 (81%). Among patients with LDL‐C not at target at 1 year, and a statin intensity registered, only a few percent received either an intensification of statin therapy or a first‐time prescription for ezetimibe within 6 months of the LDL‐C measurement (Fig. S3). For the patients included from 2016 and onwards, when ezetimibe became widely available in Sweden, less than 30% filled a prescription of ezetimibe 1 and 2 years after index ACS (Fig. S4).

Among patients without measured LDL‐C levels during the first year post‐index event, 504 (60%) filled at least one prescription for lipid‐lowering therapy (statin or ezetimibe) within the same year. For those without measured LDL‐C levels during the second and third years post‐index ACS, the number of patients who filled a prescription for lipid‐lowering therapy within those respective years was 685 (66%) and 923 (73%).

### Differences between sexes

**Fig. 2:**
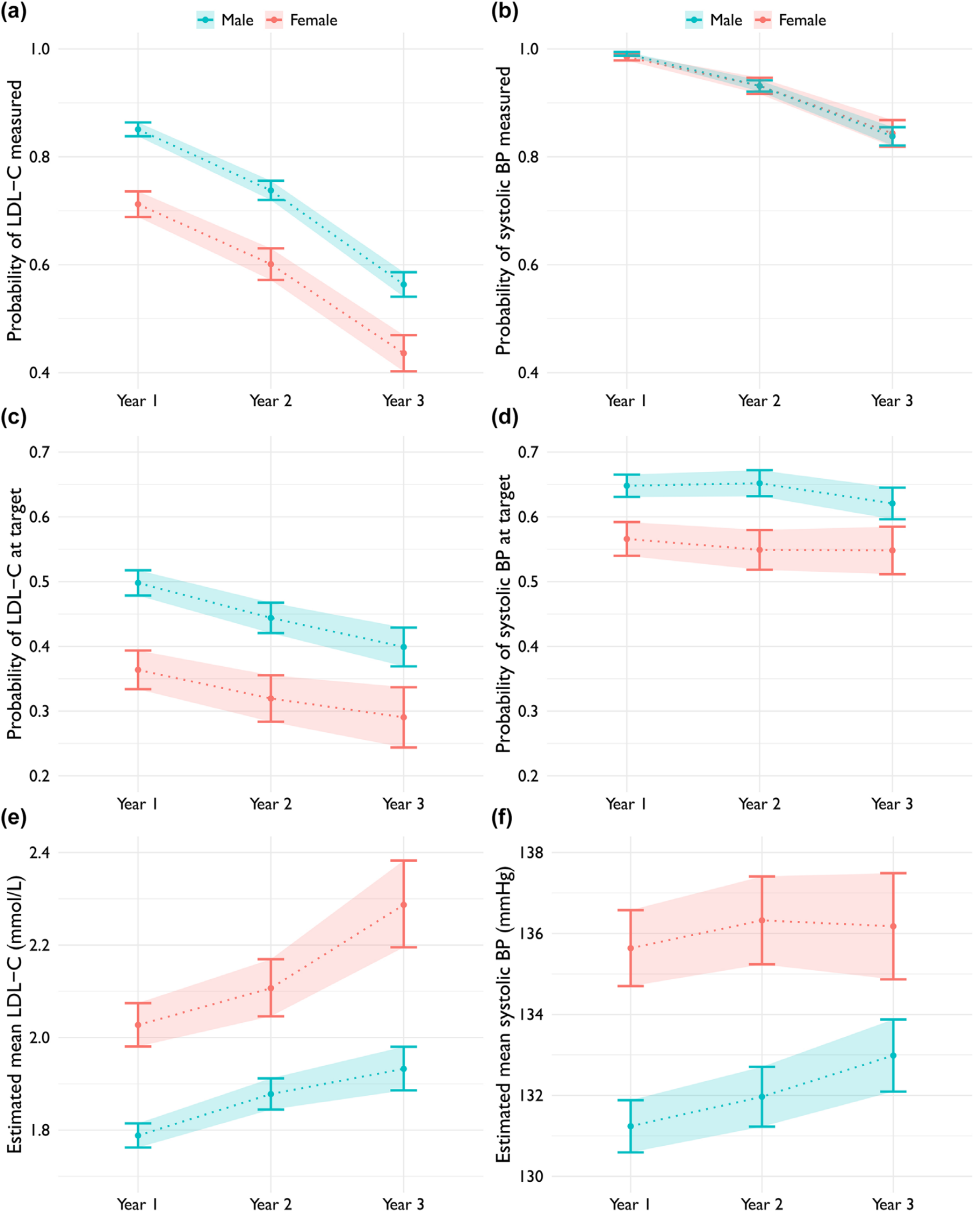
Estimated marginal probabilities of measurement (top row) of low‐density lipoprotein cholesterol (LDL‐C) (a) and systolic blood pressure (b) each year after index acute coronary syndrome (ACS) by sex. Estimated marginal probabilities of being at target (middle row) for LDL‐C (c) and systolic blood pressure (d) by sex. Estimated marginal mean levels (bottom row) of LDL‐C (e) and systolic blood pressure (f) by sex. BP, blood pressure.

The probability of having a measurement of LDL‐C any year after the index ACS was significantly lower for women, whereas for systolic blood pressure there was no difference between the sexes (Fig. 2a,b). The probability of having LDL‐C or systolic blood pressure at target was significantly lower for women, and consequently, the estimated mean levels of LDL‐C and systolic blood pressure were higher for women (Fig. 2c–f).

During the first year after ACS, the proportion of women who filled a statin prescription was 13% lower than that of men. In the second year, the difference was 14%, and in the third year, it was 18%. These differences persisted across age strata (Fig. S5) and when analysing any lipid‐lowering therapy (statin or ezetimibe, data not shown).

### Centre‐based cardiac rehabilitation

**Fig. 3:**
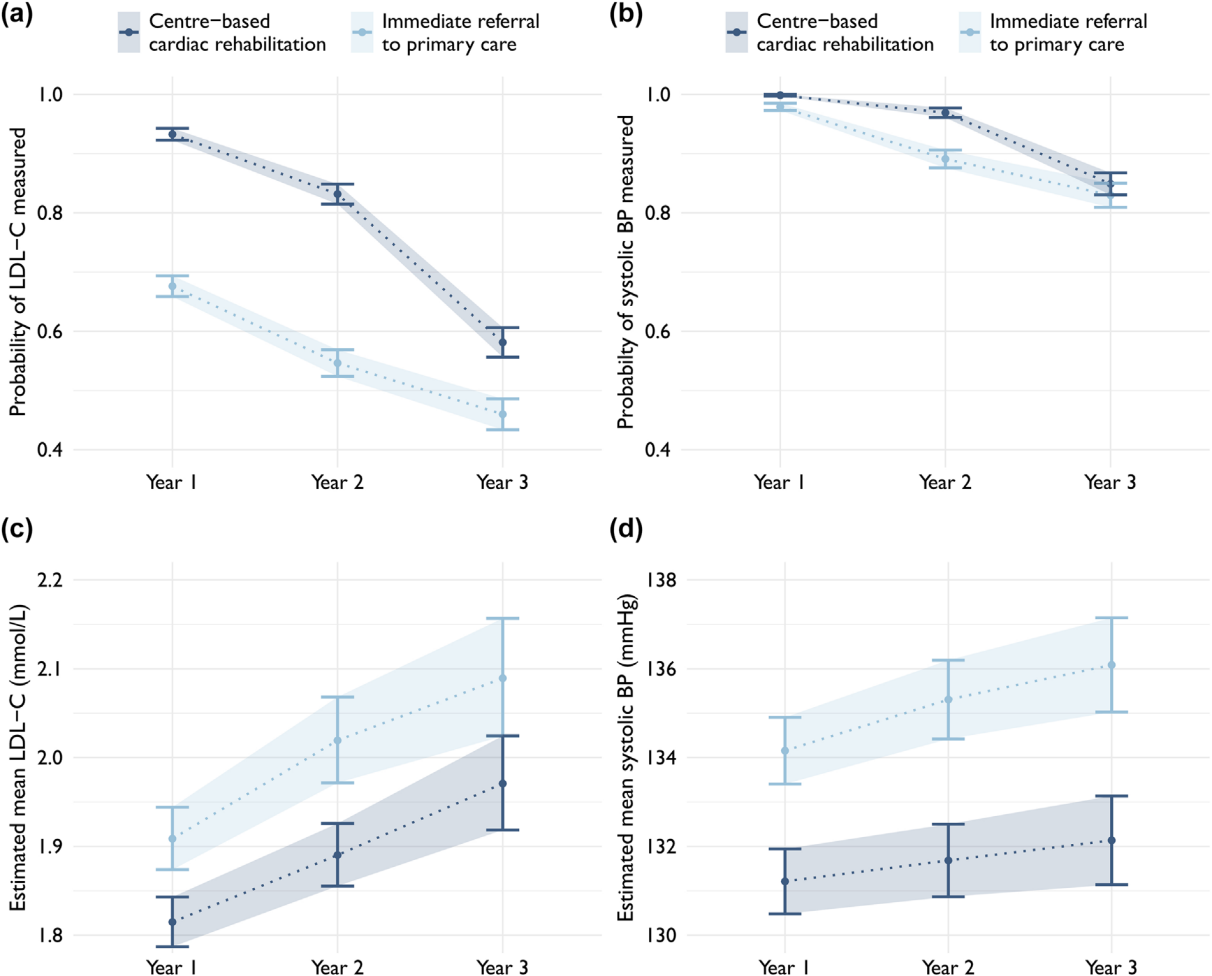
Estimated marginal probabilities of measurement (top row) of low‐density lipoprotein cholesterol (LDL‐C) (a) and systolic blood pressure (b) each year after index acute coronary syndrome (ACS) by participation in centre‐based cardiac rehabilitation, derived from logistic regression models. Estimated marginal mean levels (bottom row) of LDL‐C (c) and systolic blood pressure (d) estimated from linear regression models. BP, blood pressure.

Following the index ACS, 2214 (51%) patients participated in centre‐based CR during the first year. These patients were more likely to undergo LDL‐C and systolic blood pressure measurement and achieved lower estimated mean levels (Fig. 3). Measurement rates differed significantly for LDL‐C in all 3 years and for systolic blood pressure in the first 2 years. The association with CR participation was strongest in the first year (during centre‐based care) and decreased thereafter. Patients who participated in CR had a higher probability of reaching target systolic blood pressure than those who did not participate, whereas no difference was seen for target LDL‐C (Fig. S6a,b).

### Patients with ICD‐code denoting chronic ischaemic heart disease

**Fig. 4:**
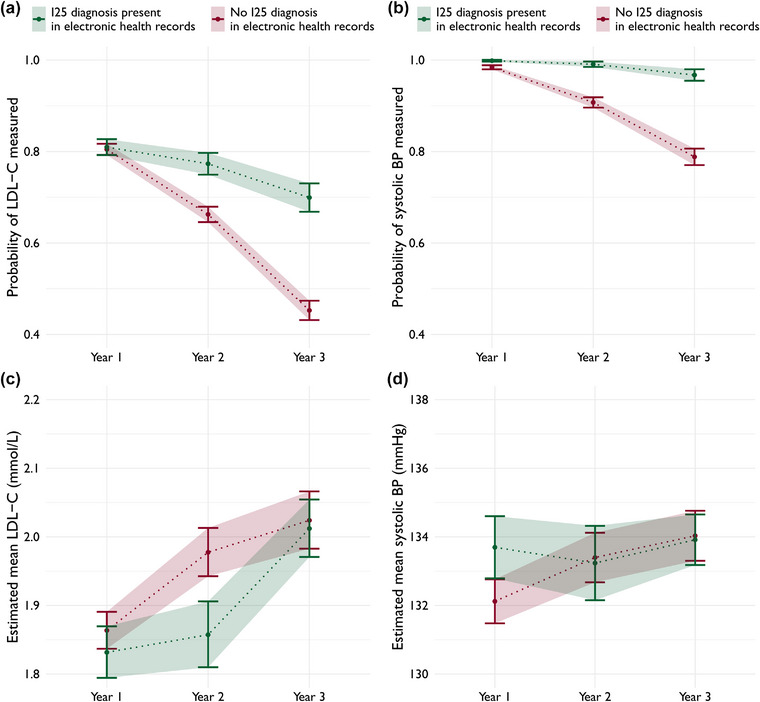
Estimated marginal probabilities of measurement (top row) of low‐density lipoprotein cholesterol (LDL‐C) (a) and systolic blood pressure (b) each year after index acute coronary syndrome (ACS) by presence of ICD‐10 diagnose code I25, derived from logistic regression models. Estimated marginal mean levels (bottom row) of LDL‐C (c) and systolic blood pressure (d) estimated from linear regression models. BP, blood pressure.

A diagnosis of chronic ischaemic heart disease (ICD‐10 code I25) was recorded in the EHR for 1440 (33%) patients during the first year after the index ACS and for 988 (29%) and 765 (29%) patients in years 2 and 3, respectively. Patients with ICD‐10 code I25 in the EHR were more likely to have LDL‐C and systolic blood pressure measured years 2 and 3 after the index event, but no differences were seen for estimated mean levels (Fig. 4). The association between having this diagnosis recorded and undergoing measurement was stronger with increasing time from the index event. However, no differences were observed in the probability of achieving target values (Fig. S6c,d). Any ICD‐10 code within I20–I25 was present in 40%, 32% and 31% of patients at years 1, 2 and 3 after the index event, respectively.

### Patients with diabetes at the time of index event

**Fig. 5:**
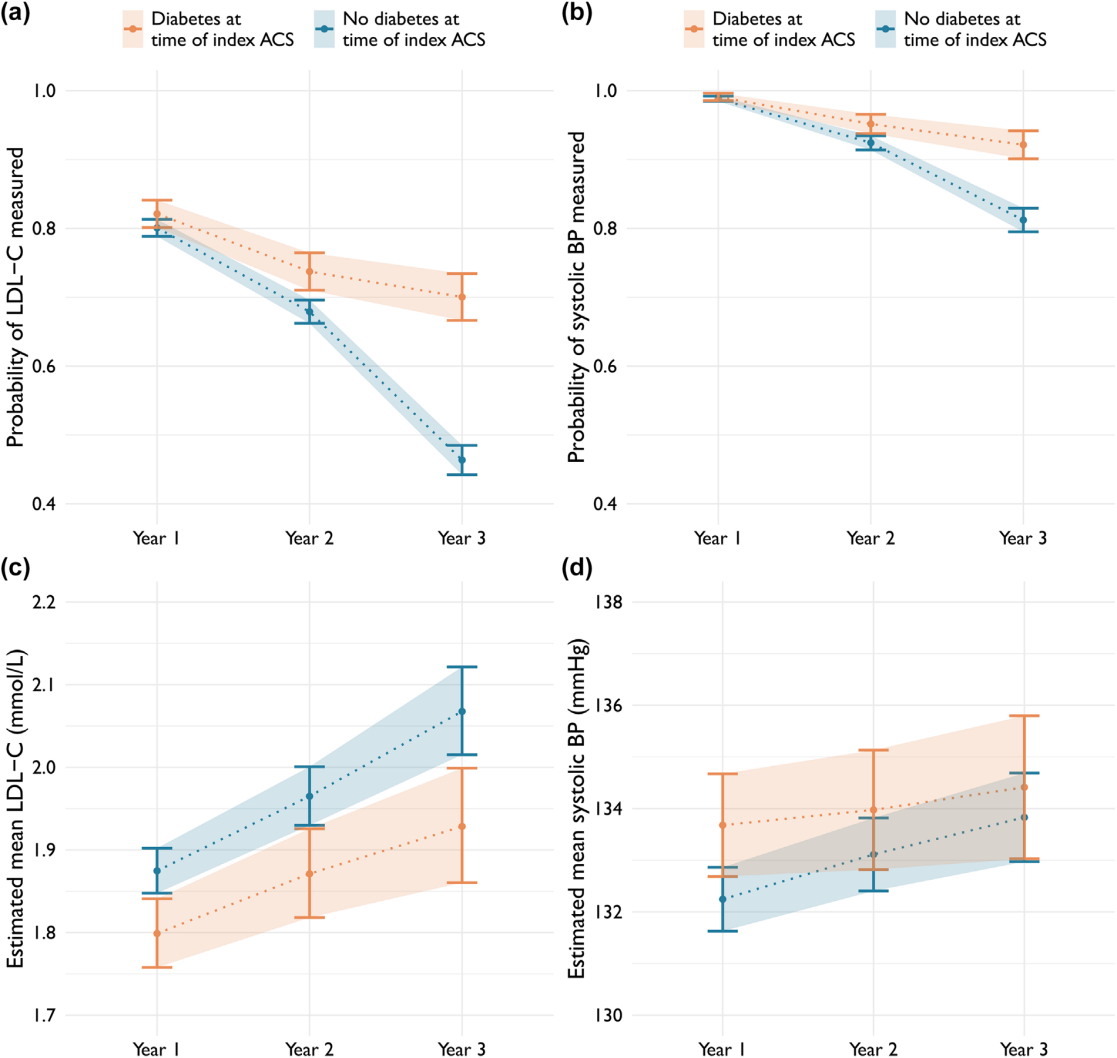
Estimated marginal probabilities of measurement (top row) of low‐density lipoprotein cholesterol (LDL‐C) (a) and systolic blood pressure (b) each year after index acute coronary syndrome (ACS) by diabetes at time of index event, derived from logistic regression models. Estimated marginal mean levels (bottom row) of LDL‐C (c) and systolic blood pressure (d) estimated from linear regression models. BP, blood pressure.

In the whole cohort, 1899 (31%) patients had diabetes at the time of index event. After the index event, follow‐up data were available for at least 1 year for 1213 (64%) of the patients with diabetes, for 2 years for 910 (48%) and for 3 years for 674 (36%). Patients with diabetes were more likely to have LDL‐C and systolic blood pressure measured years 2 and 3 after the index event (Fig. 5). As with ICD‐10 code I25, the association strengthened with greater time from the index event. Patients with diabetes had lower LDL‐C values throughout the follow‐up after ACS compared to patients without diabetes. No differences were observed for the probability of achieving LDL‐C and blood pressure targets (Fig. S6e,f).

The proportion of patients who had participated in centre‐based CR and had both diabetes and ICD‐10 code I25 in the EHR was 7% for women and 8% for men (Fig. S7).

### Sensitivity analyses

The main analyses were adjusted for covariates based on DAGs for that respective exposure and outcome (Fig. S1), meaning not all analyses were adjusted for all covariates. Adjustment for all covariates yielded nearly identical results for both probability of measurement, target achievement and the estimated mean values (Figs. S8–S10).

## Discussion

In this study, evaluating secondary prevention practices and targets during follow‐up after ACS, there are four main findings: First, target attainment was low, and both the proportion of patients meeting LDL‐C and systolic blood pressure targets and the frequency of risk factor monitoring decreased over time. Second, women were less likely than men to have LDL‐C measured, to achieve target levels for both LDL‐C and systolic blood pressure and to fill prescriptions of lipid‐lowering medication, irrespective of age. Third, patients who participated in centre‐based CR during the first year post‐ACS were more likely to have LDL‐C and systolic blood pressure measured up to 3 years after the index ACS, although the association was strongest early after the index event. Fourth, patients with a diagnosis of chronic ischaemic heart disease recorded in their EHR were more likely to undergo LDL‐C and systolic blood pressure measurements than those without such diagnosis. Similarly, patients with a diabetes diagnosis at the index event were more likely to have LDL‐C measured from the second year onwards, with this difference also increasing over time.

In this study, one fifth of patients lacked an LDL‐C measurement in the first year after index ACS, and nearly half had none by year 3. Among those with LDL‐C measured, less than half were at target in the first year and less than 40% in the third year. In a similar study of Swedish primary care patients with prior ACS, fewer than one third had annual LDL‐C measurements, whereas approximately 60% had yearly systolic blood pressure measurements [21].

Another Swedish study of more than 35,000 patients with stable coronary heart disease in primary care showed that only 18% of the patients reached target LDL‐C and 32% were not on statin treatment [22]. In the EUROASPIRE V survey carried out in 2016–2017, less than 30% had LDL‐C at target within 1.1 years after ACS [3]. By contrast, in patients attending centre‐based CR and registered in the SWEDEHEART registry in 2019, 70% of patients reached target LDL‐C 1 year after the ACS event [23]. Interestingly, CR participation was associated with better blood pressure control but not better LDL‐C target achievement. Several factors may explain this observation. Blood pressure can be modified through lifestyle interventions emphasized in CR, whereas LDL‐C reduction typically requires pharmacological intensification beyond the initial statin. The stringent LDL‐C target means that CR participants often require high‐intensity statins and ezetimibe to achieve goal, and our data suggest treatment intensification was uncommon. Additionally, blood pressure measurement was much more frequent than LDL‐C measurement, potentially enabling more responsive management. This finding suggests that the challenge in LDL‐C management is not primarily about initial education or motivation but rather about systematic treatment intensification requiring ongoing physician engagement beyond the CR programme.

A larger proportion of the patients in this study had registered measurements of systolic blood pressure through all 3 years after the index ACS, but at 3 years, half of the patients were not at target. In the EUROASPIRE survey, less than 30% of patients were at target systolic and diastolic blood pressure [3]. Although LDL‐C and systolic blood pressure frequently exceeded recommended prevention thresholds in a substantial proportion of the patients, treatment intensification was infrequent, consistent with previous reports of treatment inertia [24, 25].

Patients with prevalent diabetes mellitus at the time of ACS were more likely to have LDL‐C measured and to be at target in this study. Interestingly, in a nationwide study of more than 250,000 Swedish patients with Type 2 diabetes, those with risk factors, such as blood pressure and LDL‐C within target ranges, had a 16% lower risk of acute MI compared with matched controls [26]. This is remarkable, given that patients with diabetes mellitus are reported to have at least a two‐fold increased risk of cardiovascular disease compared to the general population. It is tempting to speculate whether this de facto reduced risk in patients with diabetes could in part be attributable to the structured standardized follow‐up offered to these patients, and whether the risk of another chronic patient group, namely, those after ACS, might also be substantially lowered with a structured life‐long follow‐up. The structured follow‐up provided to patients with diabetes in Sweden [10, 11] may explain their higher likelihood of LDL‐C measurement and the lower predicted LDL‐C levels observed in this study. Similar to participants in centre‐based CR, patients with diabetes were more likely to undergo risk factor assessments. However, in patients with diabetes, these differences became more pronounced over time, suggesting that the effect of centre‐based CR diminishes with time, whereas structured diabetes care gains increasing importance.

In women, risk factor control and lipid‐lowering therapy prescription were lower than in men, suggesting an unequal follow‐up. Similar findings have recently been published from a large primary care cohort in the south of Sweden [27].

The very low number of missing values for systolic blood pressure suggests that patients were regularly followed after their ACS. Despite this, blood pressure target achievement was low, and LDL‐C measurement and target achievement were even lower. Follow‐up in primary care after ACS is currently not structured, and many patients are missing the ICD‐10 code diagnosis of chronic coronary syndrome in the EHR.

This study is, to our knowledge, one of the first to investigate how the use of ICD codes reflects the perceived risk of future cardiovascular events. Our findings suggest that diagnostic coding practices may hold important prognostic information, underscoring the potential value of improving coding accuracy. These results point to the need for further studies—both to validate these findings and to explore whether similar patterns exist in other diagnostic areas.

There is robust data available on target achievement and prescription of secondary prevention drugs after ACS in Sweden from the SWEDEHEART registry [7, 9]. This registry, however, only covers the first year after ACS and excludes patients who were not eligible for, or did not attend centre‐based CR. In large studies from Swedish primary care regarding guideline adherence, patients are often selected based on having the ICD‐10 code I20–I25 [22, 27]. In this study, however, when identifying patients with a prior ACS diagnosis in tertiary care, 3 years after the index event only 31% of the patients had an ICD‐10 code I20–I25 registered in the current year. If patients are not receiving an ICD code for chronic coronary syndrome or previous MI, these patients are not visible in the system, and therefore, follow‐up data cannot easily be extracted, with a risk of misclassification when evaluating the care given.

A large proportion of the patients did not have LDL‐C measured; still, most of these patients still filled at least one statin prescription in the same year. Although we do not know if the statin intensity was high in all cases, this is in line with the 2013 AHA/ACC guidelines on cholesterol lowering that recommended the ‘set and forget’ approach, recommending high‐intensity statin therapy and no recommendations for specific LDL‐C goals [28]. This has, in later European and American recommendations, been replaced with a treat‐to‐target approach requiring follow‐up measurements [1, 29]. In a study of more than 95,000 Swedish patients with diabetes, refill adherence to lipid‐lowering therapy was 71%, and 56% were persistent to treatment at 3 years [30]. In that study, patients with previous cardiovascular disease had higher refill adherence and longer treatment persistence compared with those without previous cardiovascular disease.

### Strengths and limitations

All patients diagnosed with ACS in the region during the study period were included without restrictions. A unified EHR system across hospitals and primary care captured all ACS admissions, centre‐based CR participation and subsequent primary care.

The analysis of lipid‐lowering prescriptions used a simplified metric (at least one filled prescription per year) that does not capture medication adherence or treatment intensity. This metric was chosen for its descriptive utility, but more sophisticated adherence measures, such as proportion of days covered, would be needed to fully evaluate medication‐taking behaviour and its relationship to clinical outcomes. Further, only lipid‐lowering prescriptions were analysed and not antihypertensive prescriptions, because multiple antihypertensive drug classes are prescribed for various indications beyond hypertension, limiting attribution to blood pressure control. In contrast, statins are primarily prescribed for lipid management, making prescription patterns more directly interpretable.

An important quality gap was that only 64% of patients had LDL‐C measured during their index ACS hospitalization (70% within 1 week), far below the near‐universal blood pressure measurement rate (>95%). This partly reflects administrative data issues with ward transfers but more fundamentally reflects practice patterns during the study period when lipid measurement was not routine in patients above 75 years (44% of our cohort).

As an observational study, residual confounding cannot be ruled out. By clinical routine, very few patients above the age threshold for eligibility participate in centre‐based CR. This introduces a selection bias into analyses assessing participation in centre‐based CR versus no centre‐based CR, and these assessments should be assessed with caution. However, all patients should be offered participation in CR (hospital‐ or primary care‐based) and should receive equal care and achieve the same goals. Factors, such as medication adherence, smoking status, socioeconomic status, geographic distance to healthcare providers and the presence of psychiatric illness, were not accounted for.

## Conclusion

In this observational study with up to 3 years of follow‐up after ACS, patients remained engaged in care, as reflected by regular blood pressure measurements, yet LDL‐C was largely undermeasured, and targets for both LDL‐C and systolic blood pressure were frequently unmet. Women had substantially poorer post‐ACS risk factor monitoring and control than men. Indicators of a structured follow‐up, including participation in centre‐based CR and documentation of chronic ischaemic heart disease, were associated with better risk factor management, suggesting that structured follow‐up programmes similar to those for diabetes may improve secondary prevention after ACS.

## Conflict of interest statement

J.S. institutional grants and honoraria from Amgen, Arrowhead Pharma, Pfizer outside the submitted work. M.K.S. honoraria from Amgen, AstraZeneca, Boehringer Ingelheim, GSK and Novo Nordisk outside the submitted work. M.L. institutional grants and honoraria from Amarin, Amgen, AstraZeneca, Bonnier Health Care, NovoNordisk and Sanofi outside the submitted work. B.L., H.M. have no conflict of interest to disclose. E.C. institutional grants from Amgen and honoraria from Sanofi, Boehringer Ingelheim and Novartis outside the submitted work. T.C. is an employee and shareholder in Sence Research AB (an analytical company in epidemiology and biostatistics). E.H. institutional grants from Amgen, Pfizer and honoraria from Amgen, NovoNordisk, Bayer, and Astra Zeneca outside the submitted work.

## Funding information

Hjärt‐Lungfonden (grant number 20190390), and Region Uppsala

## Supporting information




**Table S1**. List of diagnoses, medications and clinical measurements and characteristics extracted from the electronical health records.
**Table S2**. Model covariates in statistical models. Comorbidities were defined as any diagnosis of chronic obstructive pulmonary disease (ICD10 code J44), heart failure (I50), stroke (I63), cancer (any C‐code), or estimated glomerular filtration rate <60 mL/min/1.73m^2^ recorded before index event discharge.
**Table S3**. Median age, interquartile range (IQR) and percentage females for patients with and without secondary preventive measurements first three years after acute coronary syndrome.
**Figure S1**. Directed acyclic graph (DAG) on confounders between different exposures: participation in cardiac rehabilitation (A), receiving ICD10 code I25 (B), having diabetes at index event (C), and sex (D) and the outcome measurement of LDL‐C. Green box is exposure, blue box with I is outcome, red boxes are measured and adjusted confounders, blue boxes are not confounders in the causal pathway and therefore not adjusted for. White circles are unmeasured confounders. The same assumptions were made for systolic blood pressure as for LDL‐C. Between the different exposures, measurement, measured value and target, some of the individual arrows differ, but the measured confounding factors were the same and therefore the same adjustment models were used.
**Figure S2**. Proportion of patients with a prior ACS event that can be followed after the event date for a minimum number of years (dark blue). The proportion of all reasons for end of follow‐up are presented with different colours.
**Figure S3**. Proportions of increase in statin intensity and initiation of ezetimibe within 6 months of an LDL‐C value not at target.
**Figure S4**. Proportions of and changes in lipid‐lowering therapy for patients from 2016 when ezetimibe became widely available. All patients (A), only male patients (B), and only female patients (C).
**Figure S5**. Proportion of patients with at least one filled statin prescription by sex and age groups during different years of follow‐up.
**Figure S6**. Estimated marginal probabilities of being at target for LDL‐C (left column) and systolic blood pressure (right column) based on centre‐based cardiac rehabilitation (top row), diagnosis of I25 (middle row), and diabetes at index event (bottom row). BP, blood pressure.
**Figure S7**. Absolute numbers and percentages of men (A) and women (B) who participated in centre‐based cardiac rehabilitation, had diabetes at index ACS and had the ICD10 code I25 any year after ACS.
**Figure S8**. Estimated marginal probabilities of measurement (top row) of LDL‐C (A) and systolic blood pressure (B) each year after index ACS by sex. Estimated marginal probabilities of being at target (middle row) for LDL‐C (C) and systolic blood pressure (D) by sex. Marginal mean levels (bottom row) of LDL‐C and systolic blood pressure by sex. Adjusted for age, previous comorbidities, prior acute coronary syndrome, previous diabetes, year of inclusion, hospital‐based cardiac rehabilitation and diagnosis of I25.
**Figure S9**. Estimated marginal probabilities of measurement (top row) of LDL‐C (A) and systolic blood pressure (B) each year after index ACS by participation in centre‐based cardiac rehabilitation. Marginal mean levels (bottom row) of LDL‐C (C) and systolic blood pressure (D). Adjusted for sex, age, previous comorbidities, prior acute coronary syndrome, previous diabetes, year of inclusion, and diagnosis of I25.
**Figure S10**. Estimated marginal probabilities of measurement (top row) of LDL‐C (A) and systolic blood pressure (B) each year after index ACS by diabetes at time of index event. Marginal mean levels (bottom row) of LDL‐C (C) and systolic blood pressure (D). Adjusted for sex, age, previous comorbidities, prior acute coronary syndrome, year of inclusion, hospital‐based cardiac rehabilitation and diagnosis of I25.

## Data Availability

Individual data from EHRs are not allowed to be shared to a third party. Access to aggregated data might be granted after review by the authors.

## References

[1] Byrne RA , Rossello X , Coughlan JJ , Barbato E , Berry C , Chieffo A , et al. 2023 ESC Guidelines for the management of acute coronary syndromes. Eur Heart J Acute Cardiovasc Care. 2023;44(38):3720–3726. 10.1093/eurheartj/ehad191 37622654

[2] Ambrosetti M , Abreu A , Corrà U , Davos CH , Hansen D , Frederix I , et al. Secondary prevention through comprehensive cardiovascular rehabilitation: from knowledge to implementation. 2020 update. A position paper from the secondary prevention and rehabilitation section of the European Association of Preventive Cardiology. Eur J Prev Cardiol. 2021;28(5):460–495. 33611446 10.1177/2047487320913379

[3] Kotseva K , De Backer G , De Bacquer D , Rydén L , Hoes A , Grobbee D , et al. Lifestyle and impact on cardiovascular risk factor control in coronary patients across 27 countries: Results from the European Society of Cardiology ESC‐EORP EUROASPIRE V registry. Eur J Prev Cardiol. 2019;26(8):824–835. 30739508 10.1177/2047487318825350

[4] Jennings CS , Kotseva K , Bassett P , Adamska A , Wood D . ASPIRE‐3‐PREVENT: a cross‐sectional survey of preventive care after a coronary event across the UK. Open Heart. 2020;7(1):e001196. 32354740 10.1136/openhrt-2019-001196 PMC7228656

[5] Ray KK , Haq I , Bilitou A , Manu MC , Burden A , Aguiar C , et al. Treatment gaps in the implementation of LDL cholesterol control among high‐ and very high‐risk patients in Europe between 2020 and 2021: the multinational observational SANTORINI study. Lancet Reg Health Eur. 2023;29:100624. 37090089 10.1016/j.lanepe.2023.100624 PMC10119631

[6] Ögmundsdottir Michelsen H , Sjölin I , Schlyter M , Hagström E , Kiessling A , Henriksson P , et al. Cardiac rehabilitation after acute myocardial infarction in Sweden—evaluation of programme characteristics and adherence to European guidelines: The perfect cardiac rehabilitation (perfect‐CR) study. Eur J Prev Cardiol. 2020;27(1):18–27. 31349776 10.1177/2047487319865729

[7] Bäck M , Leosdottir M , Hagström E , Norhammar A , Hag E , Jernberg T , et al. The SWEDEHEART secondary prevention and cardiac rehabilitation registry (SWEDEHEART CR registry). Eur Heart J Qual Care Clin Outcomes. 2021;7(5):431–437. 34097023 10.1093/ehjqcco/qcab039

[8] Leosdottir M , Hagstrom E , Hadziosmanovic N , Norhammar A , Lindahl B , Hambraeus K , et al. Temporal trends in cardiovascular risk factors, lifestyle and secondary preventive medication for patients with myocardial infarction attending cardiac rehabilitation in Sweden 2006–2019: a registry‐based cohort study. BMJ Open. 2023;13(5):e069770. 10.1136/bmjopen-2022-069770 PMC10186442 37173109

[9] Vasko P , Alfredsson J , Back M , Dahlbom L , Erlinge D , Ernkvist M , et al. SWEDEHEART Annual Report 2023. Bjorkgren I , editor. Uppsala: Uppsala Clinical Research Center; 2024.

[10] Gudbjörnsdottir S , Cederholm J , Nilsson PM , Eliasson B , Steering Committee of the Swedish National Diabetes Register . The National Diabetes Register in Sweden: an implementation of the St. Vincent declaration for quality improvement in diabetes care. Diabetes Care. 2003;26(4):1270–1276. 12663609 10.2337/diacare.26.4.1270

[11] Eliasson B , Gudbjörnsdottir S . Diabetes care–improvement through measurement. Diabetes Res Clin Pract. 2014;106(2):S291–S294. 25550056 10.1016/S0168-8227(14)70732-6

[12] Nyman U , Grubb A , Larsson A , Hansson LO , Flodin M , Nordin G , et al. The revised Lund‐Malmö GFR estimating equation outperforms MDRD and CKD‐EPI across GFR, age and BMI intervals in a large Swedish population. Clin Chem Lab Med. 2014;52(6):815–824. 24334413 10.1515/cclm-2013-0741

[13] European Association for Cardiovascular Prevention & Rehabilitation , Reiner Z , Catapano AL , De Backer G , Graham I , Taskinen MR , et al. ESC/EAS Guidelines for the management of dyslipidaemias: the task force for the management of dyslipidaemias of the European Society of Cardiology (ESC) and the European Atherosclerosis Society (EAS). Eur Heart J. 2011;32(14):1769–1818. 21712404 10.1093/eurheartj/ehr158

[14] Catapano AL , Graham I , De Backer G , Wiklund O , Chapman MJ , Drexel H , et al. 2016 ESC/EAS Guidelines for the management of dyslipidaemias. Eur Heart J. 2016;37(39):2999–3058. 27567407 10.1093/eurheartj/ehw272

[15] Mach F , Baigent C , Catapano AL , Koskinas KC , Casula M , Badimon L , et al. 2019 ESC/EAS Guidelines for the management of dyslipidaemias: lipid modification to reduce cardiovascular risk. Eur Heart J. 2020;41(1):111–188. 31504418 10.1093/eurheartj/ehz455

[16] Task Force on the Management of ST‐Segment Elevation Acute Myocardial Infarction of the European Society of Cardiology (ESC) , Steg PG , James SK , Atar D , Badano LP , Blömstrom‐Lundqvist C , et al. ESC Guidelines for the management of acute myocardial infarction in patients presenting with ST‐segment elevation. Eur Heart J. 2012;33(20):2569–2619. 22922416 10.1093/eurheartj/ehs215

[17] Williams B , Mancia G , Spiering W , Agabiti Rosei E , Azizi M , Burnier M , et al. 2018 ESC/ESH Guidelines for the management of arterial hypertension. Eur Heart J. 2018;39(33):3021–3104. 30165516 10.1093/eurheartj/ehy339

[18] Onukwugha E , Bergtold J , Jain R . A primer on marginal effects‐part II: health services research applications. Pharmacoeconomics. 2015;33(2):97–103. 25358482 10.1007/s40273-014-0224-0

[19] Norton EC , Dowd BE , Maciejewski ML . Marginal effects—quantifying the effect of changes in risk factors in logistic regression models. JAMA. 2019;321(13):1304–1305. 30848814 10.1001/jama.2019.1954

[20] Arel‐Bundock V , Greifer N , Heiss A . How to interpret statistical models using marginaleffects for R and Python. J Stat Softw. 2024;111:1–32.

[21] Bentzel S , Ljungman C , Hjerpe P , Schiöler L , Manhem K , Bengtsson Boström K , et al. Long‐term secondary prevention and outcome following acute coronary syndrome: real‐world results from the Swedish Primary Care Cardiovascular Database. Eur J Prev Cardiol. 2024;31(7):812–821. 38135289 10.1093/eurjpc/zwad389

[22] Ödesjö H , Björck S , Franzén S , Hjerpe P , Manhem K , Rosengren A , et al. Adherence to lipid‐lowering guidelines for secondary prevention and potential reduction in CVD events in Swedish primary care: a cross‐sectional study. BMJ Open. 2020;10(10):e036920. 10.1136/bmjopen-2020-036920 PMC7549446 33039993

[23] Vasko P , Alfredsson J , Back M . SWEDEHEART Annual Report 2020. Bjorkgren I , editor. Uppsala: Uppsala Clinical Research Center; 2021.

[24] Mu L , Mukamal KJ . Treatment intensification for hypertension in US ambulatory medical care. J Am Heart Assoc. 2016;5(10):e004188. 27792661 10.1161/JAHA.116.004188 PMC5121514

[25] Stenehjem K , Herren D , Pulver G , Combs B . Association of frequency of lipid testing with changes in lipid‐lowering therapy. JAMA Intern Med. 2017;177(10):1529–1531. 28846768 10.1001/jamainternmed.2017.3954 PMC5820692

[26] Rawshani A , Rawshani A , Franzén S , Sattar N , Eliasson B , Svensson AM , et al. Risk factors, mortality, and cardiovascular outcomes in patients with type 2 diabetes. N Engl J Med. 2018;379(7):633–644. 30110583 10.1056/NEJMoa1800256

[27] Bager JE , Mourtzinis G , Simons K , Rosengren A , Åberg M , Andersson T . Risk‐factor control and secondary prevention in ischemic heart disease in primary care: real‐world insights from QregPV. Eur J Prev Cardiol. 2025;zwaf052. 39919044 10.1093/eurjpc/zwaf052

[28] Stone NJ , Robinson JG , Lichtenstein AH , Bairey Merz CN , Blum CB , Eckel RH , et al. 2013 ACC/AHA Guideline on the treatment of blood cholesterol to reduce atherosclerotic cardiovascular risk in adults. Circulation. 2014;129(25_suppl_2):S1–45. 24222016 10.1161/01.cir.0000437738.63853.7a

[29] Lloyd‐Jones Donald M , Morris PB , Ballantyne CM , Birtcher KK , Covington AM , DePalma SM , et al. 2022 ACC expert consensus decision pathway on the role of nonstatin therapies for LDL‐cholesterol lowering in the management of atherosclerotic cardiovascular disease risk. J Am Coll Cardiol. 2022;80(14):1366–1418. 36031461 10.1016/j.jacc.2022.07.006

[30] Karlsson SA , Hero C , Eliasson B , Franzén S , Svensson AM , Miftaraj M , et al. Refill adherence and persistence to lipid‐lowering medicines in patients with type 2 diabetes: a nation‐wide register‐based study. Pharmacoepidemiol Drug Saf. 2017;26(10):1220–1232. 28799214 10.1002/pds.4281 PMC5656892

