# Supplementary material for: Poor long‐term cardiovascular risk factor management after acute coronary syndrome: An observational cohort study
Source: J Intern Med. 2026 Mar 20;299(6):726–40. doi: 10.1111/joim.70086 (PMC13137396; doi:10.1111/joim.70086)

Poor long-term cardiovascular risk factor management after acute coronary syndrome: An observational cohort study

Jessica Schubert, Maria K Svensson, Margret Leosdottir, Bertil Lindahl, Håkan Melhus, , Elin Täufer, Nina Johnston, Thomas Cars, Emil Hagström

Supplementary material

SUPPLEMENTARY TABLES 2
Table S1. .... *2*
Table S2. .... *2*
Table S3 .... *3*
SUPPLEMENTARY FIGURES 4
Figure S1 .... *4*
Figure S2 .... *6*
Figure S3 .... *6*
Figure S4 .... *7*
Figure S5 .... *8*
Figure S6 .... *9*
Figure S7 .... *10*
Figure S8 .... *11*
Figure S9 .... *12*
Figure S10 .... *13*

Supplementary tables

Table S1. List of diagnoses, medications and clinical measurements and characteristics extracted from the electronical health records.

**Category**	**Variables**
Demographics	Age, sex
Anthropometric measurements	Body mass index (BMI), height, weight
Cardiovascular measurements	Systolic blood pressure, diastolic blood pressure
Laboratory measurements	Serum creatinine, estimated glomerular filtration rate (eGFR), LDL-cholesterol, total cholesterol, triglycerides, HbA1c
Comorbid diagnoses	Diabetes mellitus (E10-E14), hypertension (I10.9), dyslipidemia (E78), congestive heart failure (I50), chronic obstructive pulmonary disease (J44), kidney failure (N17-N19), obesity (E66), cancer (C), tobacco use (Z72.0, F17.2)
Procedural data	Percutaneous coronary intervention (PCI), coronary artery bypass grafting (CABG) from procedural codes FNA-H, FNJ-K, or FNW
Medication dispensations	Lipid-lowering agents (ATC C10), antihypertensive medications (ATC C02, C03, C07, C08C, C09), antithrombotic agents (ATC B01), diabetes medications (ATC A10)

Table S2. Model covariates in statistical models. Comorbidities were defined as any diagnosis of chronic obstructive pulmonary disease (ICD10 code J44), heart failure (I50), stroke (I63), cancer (any C-code), or estimated glomerular filtration rate <60 mL/min/1.73m² recorded before index event discharge.

Sex	Diabetes
Age at index event	Year of inclusion
Comorbidities	Time since ACS
Prior ACS	

Table S3. Median age, interquartile range (IQR) and percentage females for patients with and without secondary preventive measurements first three years after acute coronary syndrome.

	LDL-cholesterol		Systolic blood pressure	
Patients with measurement	Median age	Percentage female	Median age	Percentage female
Year 1	69 (IQR 61-76)	28%	71 (IQR 62-79)	32%
Year 2	68 (IQR 60-74)	27%	70 (IQR 62-77)	32%
Year 3	67 (IQR 60-74)	26%	69 (IQR 61-77)	32%
Patients without measurement				
Year 1	83 (IQR 76-87)	48%	82 (IQR 66-87)	44%
Year 2	78 (IQR 68-84)	41%	76 (IQR 63-83)	32%
Year 3	72 (IQR 63-80)	37%	67 (IQR 58-75)	31%

Supplementary figures

Figure S1. Directed acyclic graph (DAG) on confounders between different exposures: participation in cardiac rehabilitation (A), receiving ICD10 code I25 (B), having diabetes at index event (C), and sex (D) and the outcome measurement of LDL-C. Green box is exposure, blue box with I is outcome, red boxes are measured and adjusted confounders, blue boxes are not confounders in the causal pathway and therefore not adjusted for. White circles are unmeasured confounders. The same assumptions were made for systolic blood pressure as for LDL-C. Between the different exposures, measurement, measured value and target, some of the individual arrows differ, but the measured confounding factors were the same and therefore the same adjustment models were used.

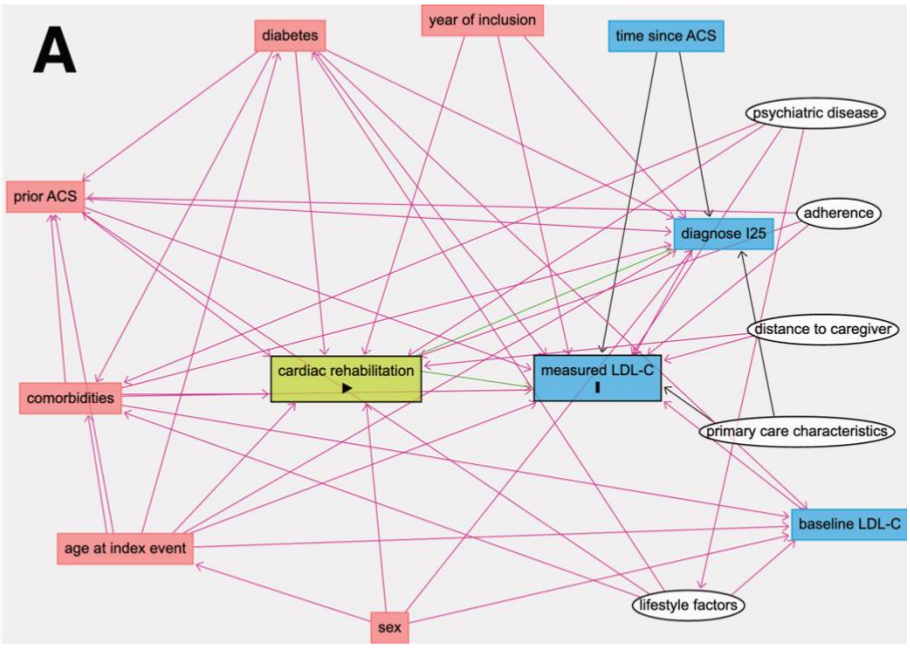

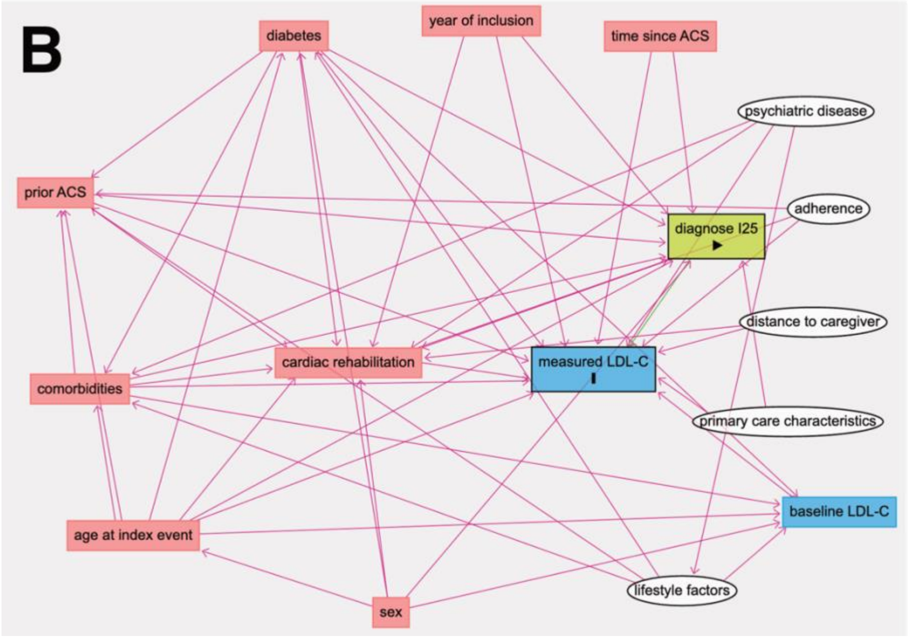

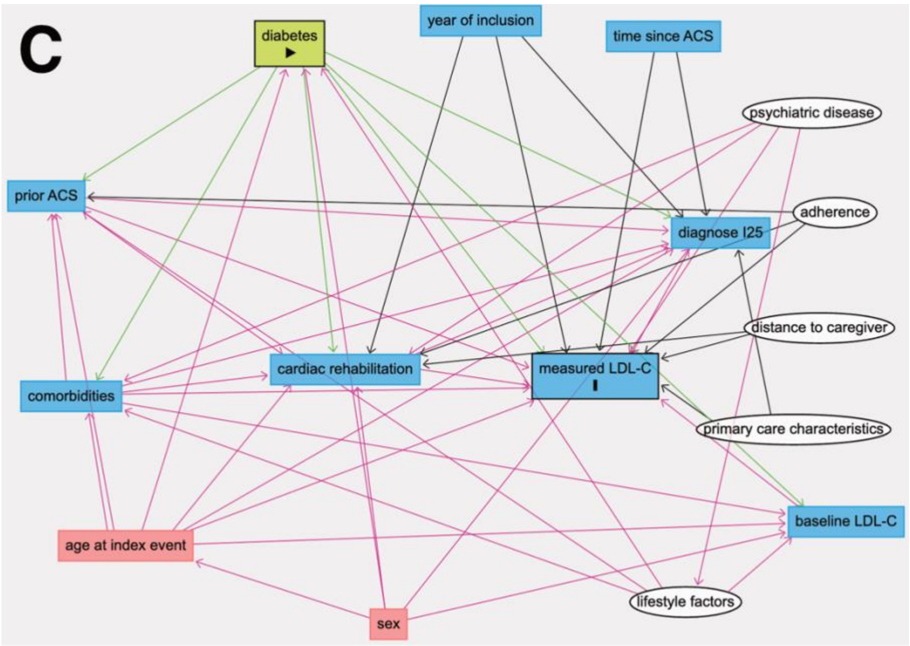
C
diabetes
year of inclusion
time since ACS
psychiatric disease
prior ACS
adherence
diagnose I25
distance to caregiver
cardiac rehabilitation
measured LDL-C
comorbidities
primary care characteristics
baseline LDL-C
age at index event
lifestyle factors
sex

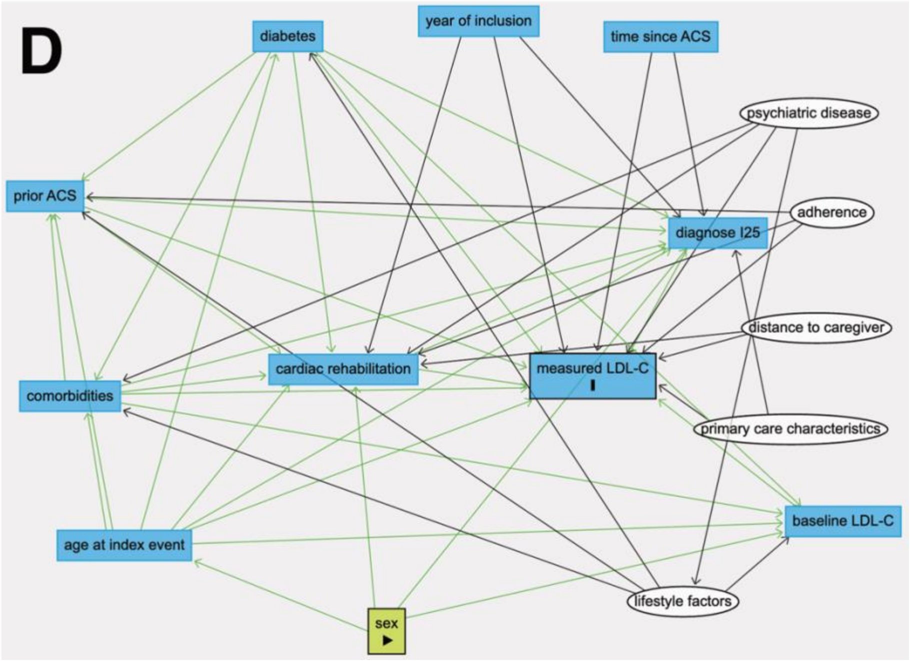
D
diabetes
year of inclusion
time since ACS
psychiatric disease
prior ACS
adherence
diagnose I25
distance to caregiver
cardiac rehabilitation
measured LDL-C
comorbidities
primary care characteristics
baseline LDL-C
age at index event
lifestyle factors
sex

Figure S2. Proportion of patients with a prior ACS event that can be followed after the event date for a minimum number of years (dark blue). The proportion of all reasons for end of follow-up are presented with different colours.

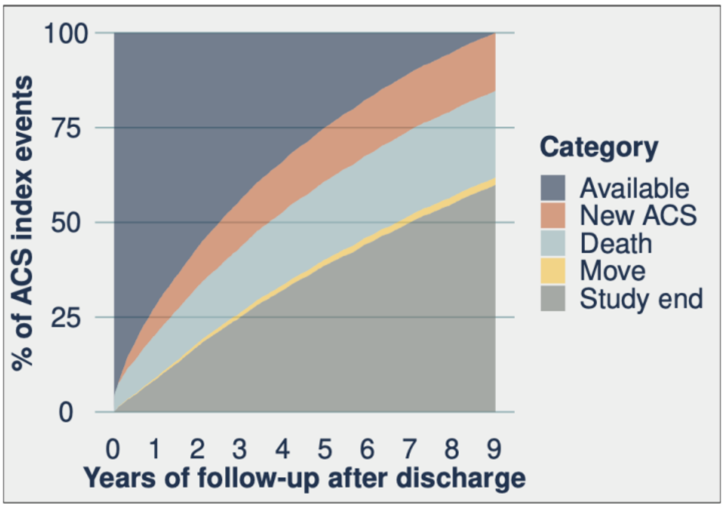

Figure S3. Proportions of increase in statin intensity and initiation of ezetimibe within 6 months of an LDL-C value not at target.

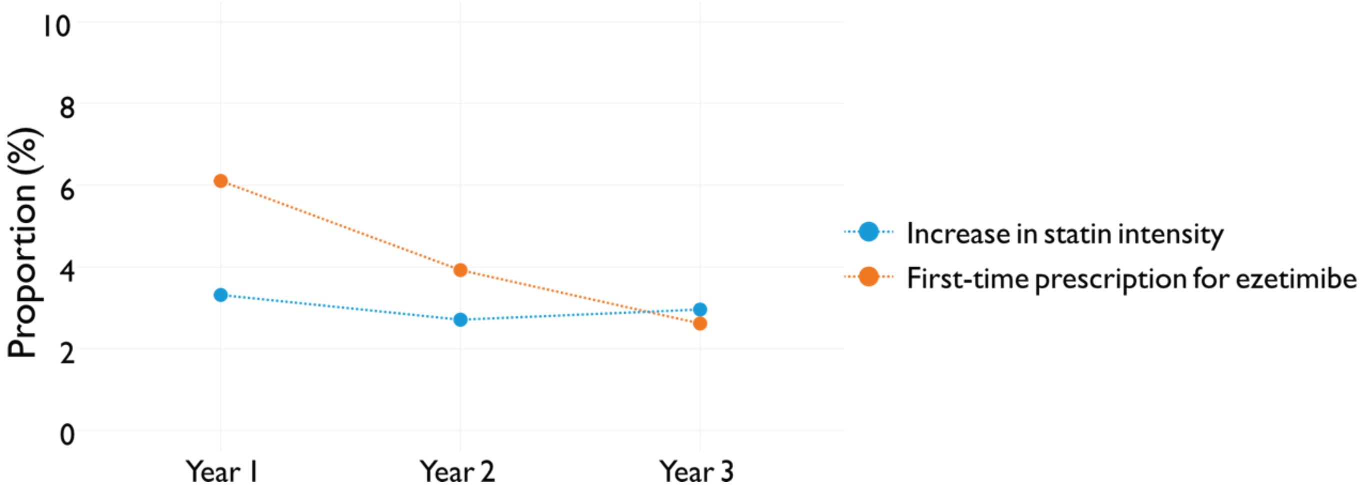

Figure S4. Proportions of and changes in lipid-lowering therapy for patients from 2016 when ezetimibe became widely available. All patients (A), only male patients (B), and only female patients (C).

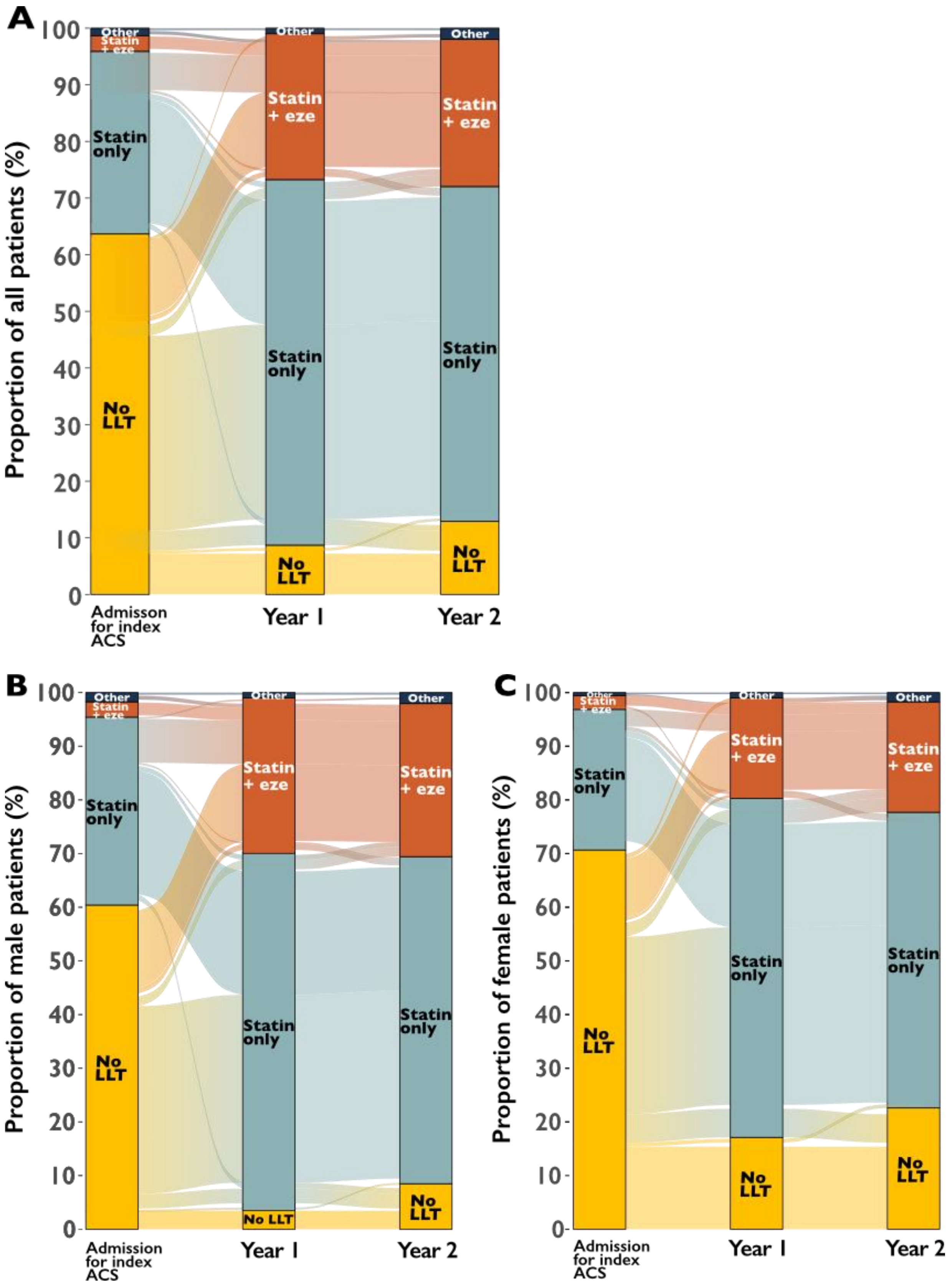

Figure S5. Proportion of patients with at least one filled statin prescription by sex and age groups during different years of follow-up.

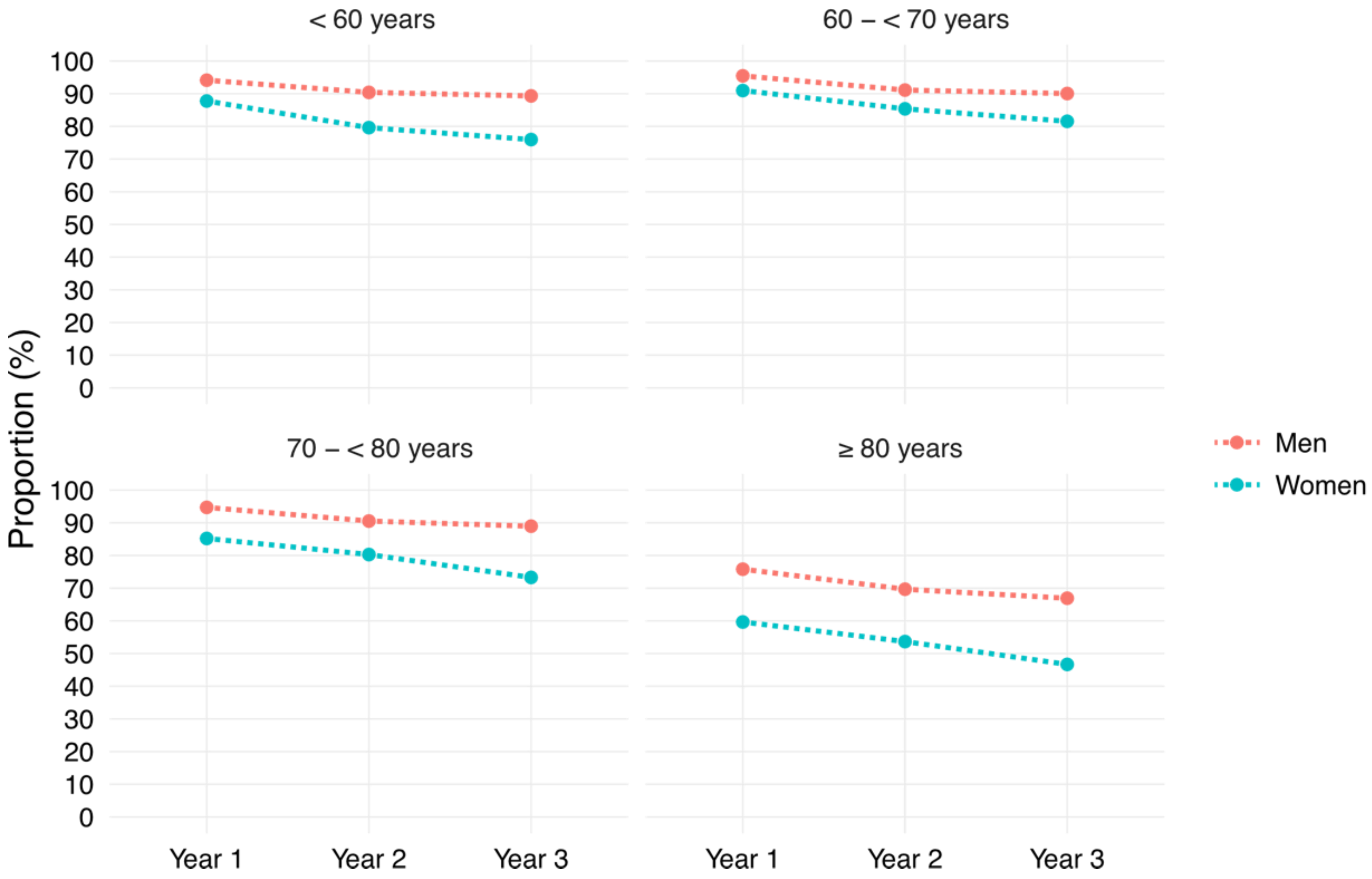

Figure S6. Estimated marginal probabilities of being at target for LDL-C (left column) and systolic blood pressure (right column) based on centre-based cardiac rehabilitation (top row), diagnosis of I25 (middle row), and diabetes at index event (bottom row). BP, blood pressure.

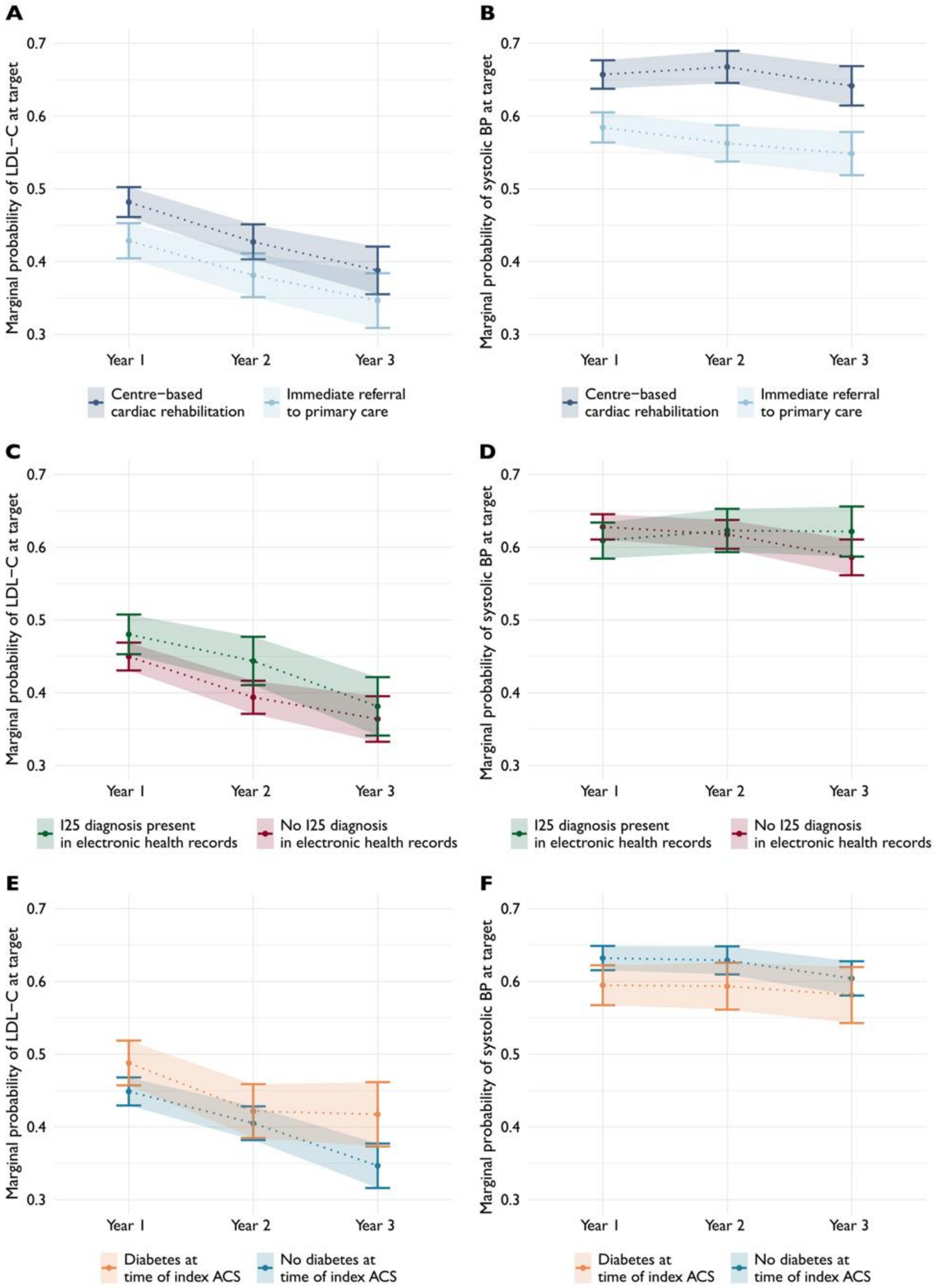

Figure S7. Absolute numbers and percentages of men (A) and women (B) who participated in centre-based cardiac rehabilitation, had diabetes at index ACS and had the ICD10 code I25 any year after ACS.

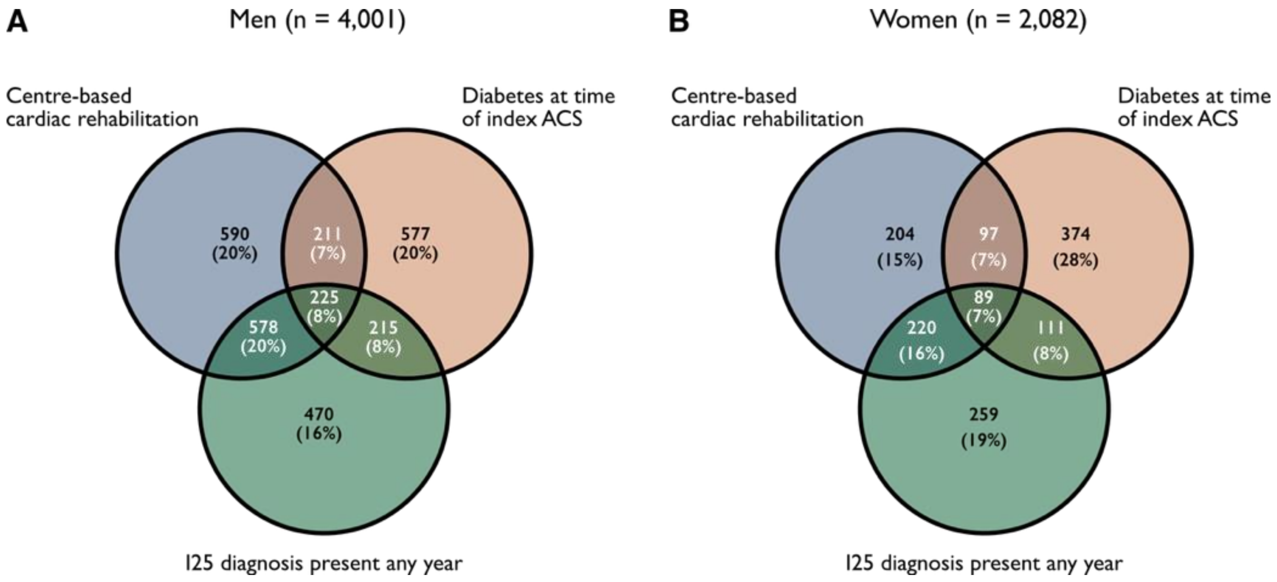

Figure S8. Estimated marginal probabilities of measurement (top row) of LDL-C (A) and systolic blood pressure (B) each year after index ACS by sex. Estimated marginal probabilities of being at target (middle row) for LDL-C (C) and systolic blood pressure (D) by sex. Marginal mean levels (bottom row) of LDL-C and systolic blood pressure by sex. Adjusted for age, previous comorbidities, prior acute coronary syndrome, previous diabetes, year of inclusion, hospital-based cardiac rehabilitation and diagnosis of I25.

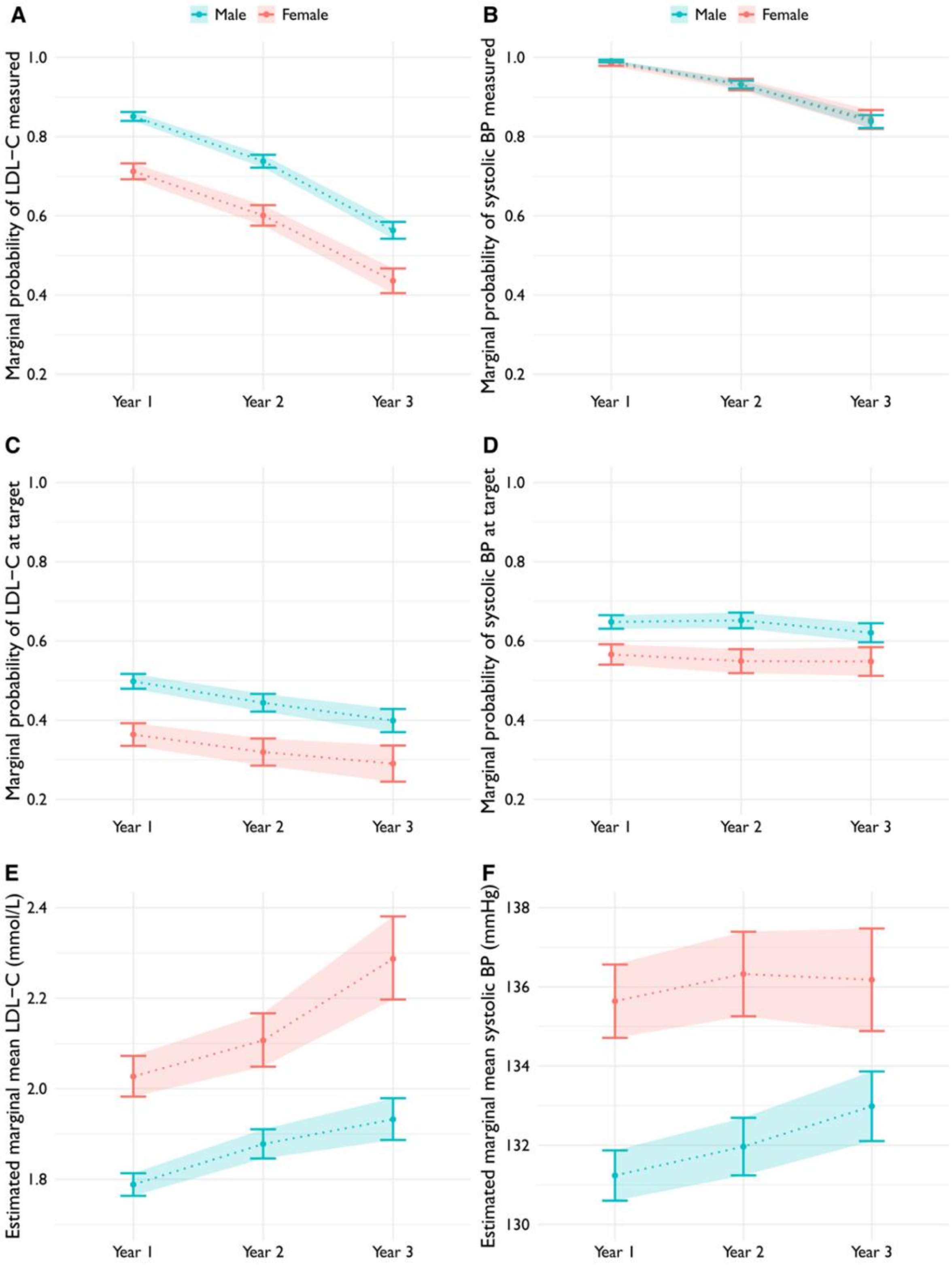

Figure S9. Estimated marginal probabilities of measurement (top row) of LDL-C (A) and systolic blood pressure (B) each year after index ACS by participation in centre-based cardiac rehabilitation. Marginal mean levels (bottom row) of LDL-C (C) and systolic blood pressure (D). Adjusted for sex, age, previous comorbidities, prior acute coronary syndrome, previous diabetes, year of inclusion, and diagnosis of I25.

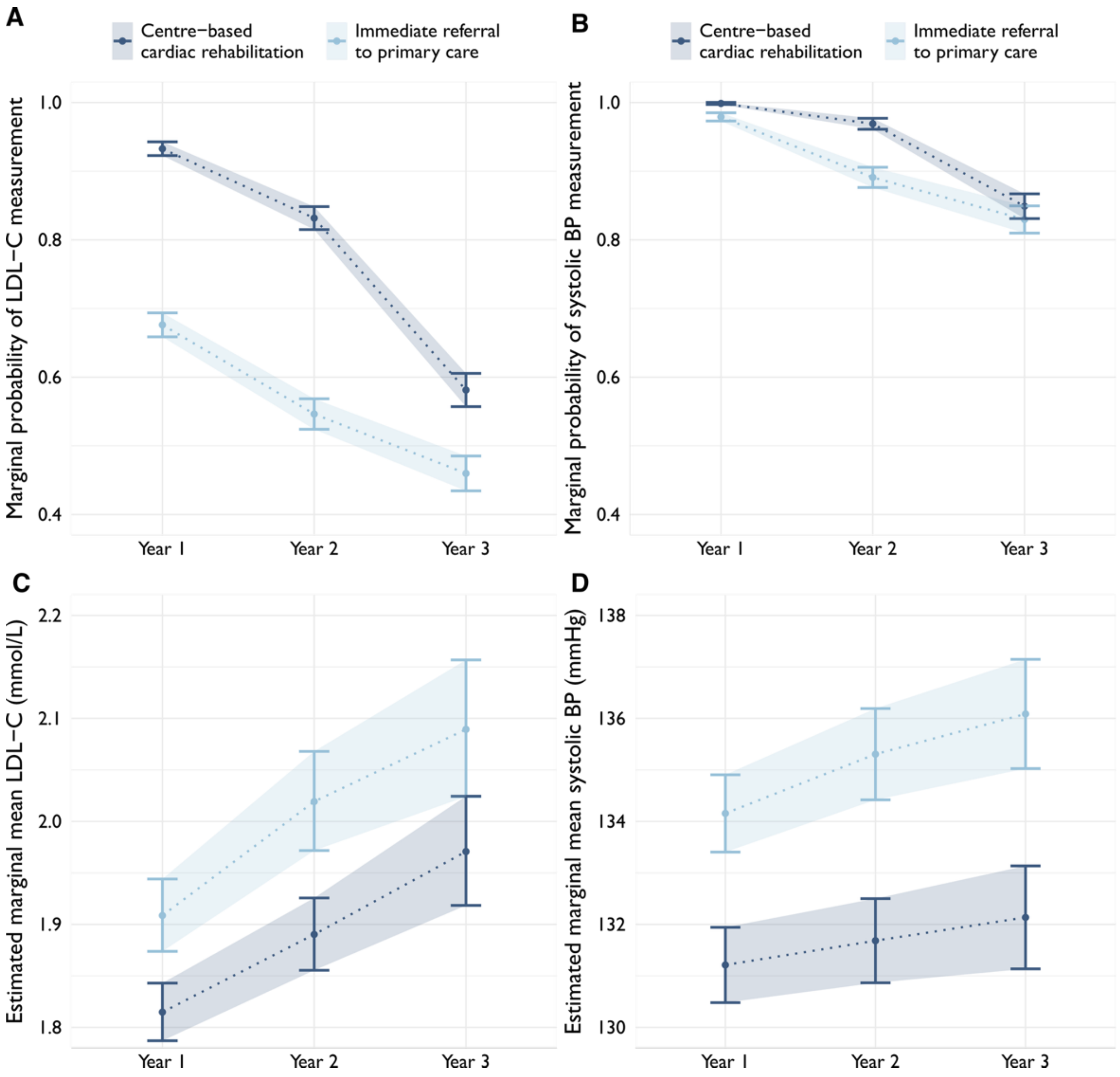

Figure S10. Estimated marginal probabilities of measurement (top row) of LDL-C (A) and systolic blood pressure (B) each year after index ACS by diabetes at time of index event. Marginal mean levels (bottom row) of LDL-C (C) and systolic blood pressure (D). Adjusted for sex, age, previous comorbidities, prior acute coronary syndrome, year of inclusion, hospital-based cardiac rehabilitation and diagnosis of I25.

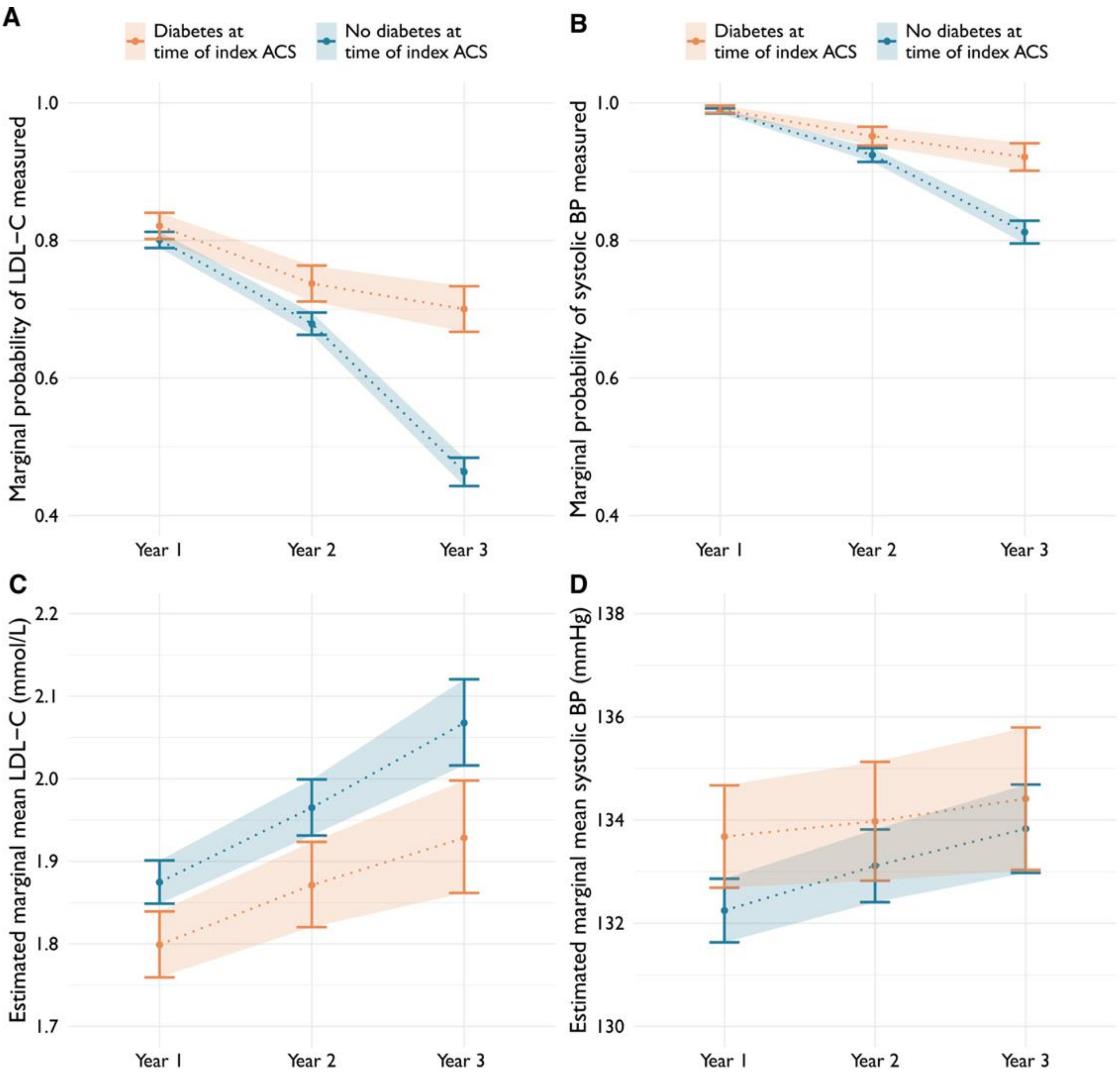

Supplement: Supplementary file 1 — Table S1. List of diagnoses, medications and clinical measurements and characteristics extracted from the electronical health records. Table S2. Model covariates in statistical models. Comorbidities were defined as any diagnosis of chronic obstructive pulmonary disease (ICD10 code J44), heart failure (I50), stroke (I63), cancer (any C‐code), or estimated glomerular filtration rate <60 mL/min/1.73m2 recorded before index event discharge. Table S3. Median age, interquartile range (IQR) and percentage females for patients with and without secondary preventive measurements first three years after acute coronary syndrome. Figure S1. Directed acyclic graph (DAG) on confounders between different exposures: participation in cardiac rehabilitation (A), receiving ICD10 code I25 (B), having diabetes at index event (C), and sex (D) and the outcome measurement of LDL‐C. Green box is exposure, blue box with I is outcome, red boxes are measured and adjusted confounders, blue boxes are not confounders in the causal pathway and therefore not adjusted for. White circles are unmeasured confounders. The same assumptions were made for systolic blood pressure as for LDL‐C. Between the different exposures, measurement, measured value and target, some of the individual arrows differ, but the measured confounding factors were the same and therefore the same adjustment models were used. Figure S2. Proportion of patients with a prior ACS event that can be followed after the event date for a minimum number of years (dark blue). The proportion of all reasons for end of follow‐up are presented with different colours. Figure S3. Proportions of increase in statin intensity and initiation of ezetimibe within 6 months of an LDL‐C value not at target. Figure S4. Proportions of and changes in lipid‐lowering therapy for patients from 2016 when ezetimibe became widely available. All patients (A), only male patients (B), and only female patients (C). Figure S5. Proportion of patients with at least one fi [file JOIM-299-726-s001.pdf]
